# A Comparative Study on Adolescents’ Health Literacy in Europe: Findings from the HBSC Study

**DOI:** 10.3390/ijerph17103543

**Published:** 2020-05-19

**Authors:** Leena Paakkari, Minna Torppa, Joanna Mazur, Zuzana Boberova, Gorden Sudeck, Michal Kalman, Olli Paakkari

**Affiliations:** 1 Faculty of Sport and Health Sciences, Research Center for Health Promotion, University of Jyväskylä, 40014 Jyväskylä, Finland; olli.paakkari@jyu.fi; 2 Department of Teacher Education, University Of Jyväskylä, 40014 Jyväskylä, Finland; minna.p.torppa@jyu.fi; 3 Department of Humanization in Medicine and Sexology, Collegium Medicum University of Zielona Gora, and Institute of Mother and Child in Warsaw, 65-729 Zielona Góra, Poland; joanna.mazur@imid.med.pl; 4 Institute of Biology and Ecology, Faculty of Science, Pavol Jozef Safarik University, 040 01 Košice, Slovakia; zuzana.boberova@upjs.sk; 5 Institute of Sport Science, Education and Health Research, Eberhard Karls University Tübingen, 72074 Tübingen, Germany; gorden.sudeck@uni-tuebingen.de; 6 Department of Recreation and Leisure Studies, Faculty of Physical Culture, Palacky University Olomouc, 779 00 Olomouc, Czechia; michal.kalman@upol.cz

**Keywords:** health literacy, adolescent, self-rated health, comparative study

## Abstract

(1) Background: There is a need for studies on population-level health literacy (HL) to identify the current state of HL within and between countries. We report comparative findings from 10 European countries (Austria, Belgium (Fl), Czechia, England, Estonia, Finland, Germany, Macedonia, Poland, and Slovakia) on adolescents’ HL and its associations with gender, family affluence (FAS), and self-rated health (SRH). (2) Methods: Representative data (N = 14,590; age 15) were drawn from the HBSC (Health Behavior in School-Aged Children) study. The associations between HL, gender, FAS, and SRH were examined via path models. (3) Results: The countries exhibited differences in HL means and in the range of scores within countries. Positive associations were found between FAS and HL, and between HL and SRH in each country. Gender was associated with differences in HL in only three countries. HL acted as a mediator between gender and SRH in four countries, and between FAS and SRH in each country. (4) Conclusions: The findings confirm that there are differences in HL levels within and between European countries, and that HL does contribute to differences in SRH. HL should be taken into account when devising evidence-informed policies and interventions to promote the health of adolescents.

## 1. Introduction

In its most recent policy document, the World Health Organization [1] has emphasized the need to monitor health literacy (HL) in order to identify the current state of HL in various population groups. This will provide a starting point for data-driven policies, and for practical interventions targeted at HL development. HL has been recognized as a critical determinant of health, and as a contributory factor to empowerment and equity [2]. 

HL has been defined [3] as a competence to “gain access to, understand and use information in ways which promote and maintain good health.” As applied to adolescents, it explains differences in health and serves as a mediator between several structural stratifiers and health indicators [4]. Since HL can be developed, it can undoubtedly act as a way of addressing adolescents’ health disparities, which tend to track forward to adulthood [5]. It is also linked to power gained through knowledge [6], with a potential for empowerment and autonomy [1]. 

So far, there have been relatively few comparative studies on population-level HL in various countries. One of the few studies in the field is that of Sorensen and colleagues [7], who compared adults’ HL in eight different European countries (mean age 43.3–48.7 [8]). They found that HL levels differed between countries and subgroups. Weak health literacy (i.e., in the limited category) varied from 28.7% (Netherlands) to 62.1% (Bulgaria). This category was more prevalent among people with low affluence and those with a lower level of education, among men and older people, and among people with a greater use of healthcare services. The study showed that HL deficit is a definite problem in Europe, and it confirmed that some subgroups were more likely than others to exhibit an unacceptably low level of HL.

The importance of HL in understanding health disparities has been acknowledged [9]. However, not much is known about how it might explain differences in adolescents’ health or how it might mediate between various structural stratifiers and health indicators (such as self-rated health) in various countries using a comparative approach. Nationally conducted studies have shown that higher HL is more common among various categories, including girls [10], children from more affluent families [10,11,12], those with higher school achievement [10,12], and those with the motivation to learn health issues [11]. Furthermore, higher HL has been associated with better self-rated health [13,14]. There are clear public health issues involved in understanding how differences in self-rated health operate, as either a subjective indicator of general health, or as a robust measure of it [15,16]. Self-rated health has been linked to a number of phenomena, including mortality and morbidity, functional ability, and physical and mental symptoms [17]. Studies have shown that certain factors—such as one’s childhood socioeconomic position—appear to explain adolescents’ self-rated health independently, and continue to do so later in their lives [18]. 

In a longitudinal study, Bauldry et al. [18] found that certain factors, including various risk behaviors, mediated the relationship between background factors and self-rated health throughout the follow-up years. They concluded that, as early as possible, efforts should be targeted at those mediating mechanisms that generate disparities in health. It would be reasonable to expect that HL in particular would act as a mediator between background factors (such as family affluence or gender) and health (self-rated or objectively measured). So far, no longitudinal studies have addressed this directly, but in a cross-sectional study, Paakkari et al. [4] were able to show that adolescents’ health literacy did indeed serve as a mediator between family affluence and self-rated health. The link between poor self-rated health and low HL has also been detected among adults [7,12,19].

**Figure 1:**
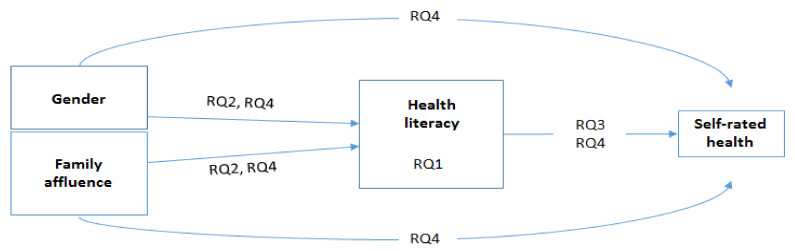
A path model explaining the associations between gender, family affluence, health literacy, and self-rated health.

The aim of this paper is to report the first comparative findings from 10 European countries, using data from the Health Behavior in School-aged Children (HBSC) survey. The more specific research questions for this study were (see also Figure 1)

- Are there differences in adolescents’ health literacy in 10 European countries? (RQ1)
- Are (a) gender and (b) family affluence associated with health literacy in the 10 participating countries? (RQ2)
- Does health literacy explain self-rated health in the 10 participating countries? (RQ3)
- Does health literacy mediate the association between gender and self-rated health, or between family affluence and self-rated health, in the 10 participating countries? (RQ4)

## 2. Materials and Methods

### 2.1. Sample and Procedure

Cross-sectional data from the *Health Behavior in School-Aged Children* (HBSC) study (collected in 2017–2018) were used. HBSC is a collaborative study with World Health Organization. The data for it are collected every four years using a standardized research protocol in all participating countries, meaning that all the countries share the same mandatory questions and follow the same sampling and data collection procedures. In addition, each country may select optional questions to be included within its national survey. This paper reports the findings from the 10 countries (Austria, Belgium (Fl), Czechia, England, Estonia, Finland, Germany, Macedonia, Poland, and Slovakia) who selected the Health Literacy for School-Aged Children (HLSAC) instrument as an optional package for inclusion in their national survey. The data used in this study consisted of, in total, 14,590 15-year-old pupils (boys: *N* = 7087; girls: *N* = 7503).

Each country obtained ethical approval, and depending on the country, active or passive consent was asked from the students and their guardians. Participation in the study was entirely voluntary and anonymous. More detailed information on the methodology of the HBSC study can be found elsewhere [20].

### 2.2. Measures

Self-reported *gender* and an indirect measure of family wealth (*family affluence*, FAS III [21]) were used as independent variables. FAS III (six items) includes questions on number of cars, having one’s own bedroom, number of computers, number of bathrooms, having a dishwasher, and number of family vacations abroad. In line with Inchley et al. [22] (p. 17), the scale was applied “to assess the relative affluence of the families by comparing the individual’s summary score from the FAS to all other scores in the respective country/region” and “to identify groups of young people in the lowest 20% (low affluence), middle 60% (medium affluence) and highest 20% (high affluence).” The FAS variable with three categories was used in the analyses.

**Figure 2:**
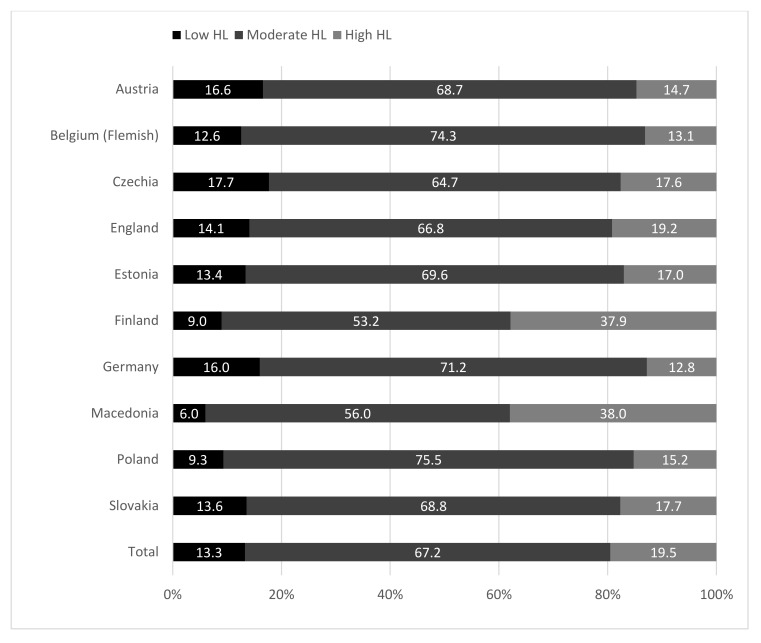
Levels of health literacy; by country and for the total sample (percentage distributions).

A brief 10-item Health Literacy for School-Aged Children (HLSAC) instrument, validated for 13 and 15-year olds [14,23] was used to measure the adolescents’ subjective (self-reported) HL. HLSAC was developed on an understanding of HL as a set of competencies to promote and sustain health, and to identify and influence the factors that affect health. The items focused on five competence areas (two items each): theoretical knowledge, practical knowledge or skills, critical thinking, self-awareness, and citizenship. All the items took the form *I am confident that…* and the response options were *(1) not at all true, (2) not quite true, (3) somewhat true,* and *(4) absolutely true*. A sum-score was generated from the responses to the 10 items; hence the range in total was 10–40. This continuous variable was used in all analyses. In addition, for descriptive purposes, the levels of HL were classified into three groups with scores 10–25 indicating *low* HL, scores 26–35 *moderate* HL, and scores 36–40 *high* HL [10] (see Figure 2). Findings on the reliability and validity of the scale are thoroughly reported in our previous work [14,23]. Cronbach alpha is reported to be included in cross-national data .85 [14] (Paakkari et al., 2019). *Self-rated health* (SRH) was used as a dependent variable, and was measured by a single item [24]; here the question took the form *Would you say your health is…* and the response options were *poor, fair, good,* and *excellent.*

### 2.3. Statistical Analysis

The differences in HL between the countries were inspected via a univariate analysis of variance (with Bonferroni correction for pairwise comparisons). Research questions 2–4 were examined using path modelling (Figure 1) with the maximum likelihood estimator. The hierarchical structure of the sample (children nested in classrooms) was taken into account by correcting the standard error estimates as necessary, using the COMPLEX option provided by Mplus (version 8.3). 

## 3. Results

### 3.1. Differences in HL Level (RQ1)

A univariate analysis of variance (ANOVA) showed statistically significant differences between countries (*F*(9) = 70.19, *p* = 0.000). The highest HL mean scores were obtained in Macedonia (33.93) and Finland (32.81), and the lowest in Austria (29.88) and Germany (29.99). According to the pairwise comparisons, both Macedonia and Finland had statistically significantly higher mean scores than any of the other countries (Table 1). 

Regarding the three levels of HL (low, moderate, high), in total, 13% of the participants had low HL, 67% had moderate HL, and 20% reached a high level of HL (Figure 2). The differences between the countries were significant (*X*^2^(18) = 760.75, *p* < 0.001). The proportion of low HL among the respondents varied from 6.0% (Macedonia) to 17.7% (Czechia), and the proportions of high HL varied from 12.8% (Germany) to 38% (Macedonia).

### 3.2. Associations of Gender and Family Affluence with HL, and of HL with Self-Rated Health (RQ2–3)

Table 2 shows the correlation coefficients between the measures for each country, and Supplementary Materials File 1 reports the descriptive statistics of gender, FAS, and SRH. Model 1 examined whether FAS and gender explained the variance in HL. It suggested significant associations between FAS and HL in each country, with higher FAS being associated with higher HL. The standardized path estimates varied between 0.09 and 0.16, suggesting that 0.8–2.6% of the HL variance was explained by FAS. By contrast, gender was significantly associated with differences in HL (with girls showing higher HL) only in Estonia (β = 0.10, R^2^ = 1%), Macedonia (βn= 0.06, R^2^ = 3.6%), and Poland (β = 0.10, R^2^ = 1%).

Next, Model 2 was constructed to estimate the effect of HL on SRH (RQ3). A significant (*p* < 0.001) positive association was found in each country. The path estimates varied between 0.12 and 0.27, suggesting that 1.4–7.3% of the SRH variance was explained by HL.

### 3.3. HL as a Mediator between Gender, Family Affluence, and Self-Rated Health (RQ4)

In order to examine the mediating effect of HL, we first fitted a model in which gender and FAS explained variance in SRH directly (Table 3, Models 3 and 4). In the final model (Model 5) with HL as mediator the direct gender effect on SRH remained significant in all the participating countries, explaining 1.2–3.2% of the SRH variance. Boys had slightly higher SRH than girls (expressed as minus scores in Table 3). The direct effects of FAS on SRH remained, and were partially mediated by HL. FAS explained 0.1–2.0% of the variance, and was significant in six countries (Belgium, Czechia, England, Estonia, Finland, and Germany). 

Finally, indirect effects were examined (with HL as a mediator between gender/FAS and SRH). A significant indirect effect from gender to SRH through HL was found in Estonia, Finland, Macedonia, and Poland. There was also a positive indirect effect from FAS to SRH through HL in each participant country; however, the indirect effects were weak.

## 4. Discussion

The HBSC study reported here was the first study to obtain comparative findings on the HL levels of 15-year-old adolescents in Europe, and the associations of HL with self-rated health. 

The findings showed differences in adolescents’ HL in Europe, with Macedonia and Finland having the highest proportion of adolescents reporting high HL, and the smallest proportion of adolescents reporting low HL. In general, most of the adolescents in all the participating countries had a moderate level of HL, but in each country, there were substantial proportions of adolescents with low HL. Low HL was most common in Czechia (17.7%) and least common in Macedonia (6.0%). Nevertheless, many adolescents exhibited high HL, with this category occurring most frequently in Macedonia (38.0%) and Finland (37.9%), and least frequently in Germany (12.8%). 

HL explained the differences in self-rated health in all the countries. In several countries, it also served as a link between gender and self-rated health, and between family affluence and self-rated health. When these findings are linked to the findings showing clear differences in HL between and within countries, one can identify a definite public health and health promotion issue in the extremes of low and high HL encountered. Similar findings on the association between HL and self-rated health have been reported previously [13], also among adult populations [7,19]. 

The findings also confirmed that family affluence is as a predictor of HL [11,12,25,26]. It is notable that gender was associated with differences in HL levels (with girls showing higher HL) in only three countries (Estonia, Macedonia, and Poland), whereas family affluence explained the HL differences (the higher the FAS, the higher the HL) in all the countries. However, due to the fact that no other structural stratifiers were studied, we do not know how far they would explain variations in HL levels if factors such as school achievement and educational orientation [7,27,28], or age and ethnicity [29] were added to the model. 

Whatever factors one adds to the model, there is little doubt that the level of equity in HL will remain an important question. Here it is worth noting that socioeconomic status has been found to be a determinant of equity also in competencies other than HL. Thus, in 2015, socioeconomic status was found to explain 19% of the variation in the PISA (Programme for International Student Assessment) science performance in Belgium and Czechia, 16% in Slovakia, Germany, and Austria, and 10% in Finland [30].

There has been critical discussion on the nature of the bias that might link self-rated health to self-rated HL. Ruegg and Abel [31] (p. 544) have argued that “a positive association might occur due to general optimism (or pessimism) that affects self-rated health and self-rated HL in the same way.” Paakkari et al. [4] also noticed that subjective (self-rated) HL provided a better explanation of a range of perceived health indicators (including self-rated health) than did health behaviors; they speculated that various self-perceived indicators might tend to group together, even though as concepts and phenomena they were not fully equivalent. This perspective on possible bias definitely requires further exploration. Nevertheless, extensive research on the importance of self-rated health as an indicator of health, morbidity, and mortality underlines the importance of identifying and targeting all the factors that could explain self-rated health, including self-rated HL. As Broder and colleagues [32] have argued, “failing to provide young populations with health literacy and health-promoting capacities would constitute an increased risk for the individual and society in terms of poorer health outcomes and higher costs.”

According to Rowlands et al. [33], 19 European countries are addressing low HL as a public health issue and have already targeted, or are about to target, HL in their policies. However, only a few countries have included HL within their national school curricula [34], even though this is an important means to reach all school-aged children [2]. The higher HL among Finnish adolescents as compared to eight other European countries could be partly explained by the fact that HL competencies are taught in Finnish schools through an independent and obligatory school subject called health education. The positive influence of school-based health promotion and education programs on pupils’ HL level has also been identified elsewhere [12]. On the other hand, no similar explanation can be offered regarding the high HL of Macedonian adolescents. Furthermore, Macedonia was not among the countries that had reported having HL in their political agenda, in contrast with the UK, Austria, Czechia, Germany, and Belgium [33]. This would indicate a need to study further the fairly high HL mean score in Macedonia. It should be noted, however, that in some countries (such as Germany) HL has only recently found its place on the political agenda, i.e., in the last 5–10 years [35]. Hence, it may be that the possible impact on the HL of adolescents has yet to emerge. In any case, at the present time, no detailed information is available on the existence of population-based policies and practices focusing on adolescents in the participating countries. 

Though Finland was among the countries with the highest average HL level and the smallest proportion of adolescents with low HL, the country also showed the widest extremes in HL levels. This finding contrasts that of Sorensen et al. [7], who discovered that the standard deviation was larger in countries where the average HL levels were lower. They concluded that this “lower HL on average, [with] wider standard deviation” in these countries might indicate “more inequality in terms of the distribution of health literacy in their population” (p. 1055). Further research is needed on the issue. 

It would be interesting to examine whether the numerical grade in health education as a school subject explains subjective HL in Finland—by analogy with the situation previously found among adolescents, namely that the lower the grades in mathematics and the first language, the lower the HL level [10]. As no other participating country has a similar school subject, or similar health education grading, the possible association between grades and self-perceptions could partly explain the wider range of scores found in Finland. On the other hand, one might ask whether education in health issues could actually increase differences in HL. Research up to now has indicated that schooling decreases cognitive gaps as compared to non-schooling experiences [36], with the implication that education in health issues would be beneficial in every possible respect [6]. However, we also know that some adolescents learn better than others, which would mean that even if average HL increases through education, the deviation between high and low performers could become larger. 

There is a need for more research to understand the differences in health literacy levels within and between the countries, such as why gender explains variance in HL in some countries but not in others. One would also wish to analyze further the competencies (items) which demonstrate differences, in order to understand better the differences that are most relevant to (for example) planning HL interventions. Research is also needed on objectively measured health literacy and its relation to a range of health indicators, with the use also of longitudinal studies to determine (i) how HL tracks forward to adulthood, and (ii) the role it plays as an intermediary between various background factors and health. Furthermore, critical discussion is needed on the size of the coefficient of determination, HL, and health inequalities: how big *should* or even *could* the coefficient of determination be to define HL as a “critical” or “important” determinant of health? Though countries varied, in the present study HL explained up to 7% of the variation in self-rated health, but more than gender or FAS. A previous study of Finnish adolescents showed that HL explained 10% of the variation, and more than gender, FAS school achievement, educational orientation, or age, which together with HL explained 12% [4]. Few other studies have shown findings of a similar size range [13,14] even if a study on adult samples reported stronger effects [37]. In any case, bearing in mind also that self-rated health is a robust predictor of mortality [38], it can be suggested that any factor that contributes to a decrease in health disparities is important, including HL.

## 5. Conclusions

This study offers the first comparative findings on adolescents’ health literacy levels in the European region. Despite the limited number of factors studied, the findings do appear to support Trezona et al. [9] in viewing health literacy as “a convenient way of describing a measurable variable that can be used to understand and explain variation in health.” One can anticipate that the findings of this research together with suggestions for future research, will lead to a fuller understanding of the current status of health literacy among adolescents in Europe, with possibilities for more evidence-informed policies in the region.

## Figures and Tables

**Table 1:** Descriptive statistics and multiple comparisons of health literacy; by country and for the total sample.

Country	N	Mean	SD	Skewness	Kurtosis
Austria	1190	29.88 ^5,6,8,9,10^	5.32	−0.53	1.52
Belgium (Flemish)	1374	30.33 ^6,8^	4.73	−0.58	1.24
Czechia	3154	30.06 ^6,8,9,10^	5.72	−0.51	0.90
England	710	30.57 ^6,8^	5.57	−0.57	0.99
Estonia	1506	30.54 ^1,6,8^	4.98	−0.55	1.04
Finland	993	32.81 ^1,2,3,4,5,7,9,10^	6.33	−1.15	2.13
Germany	1429	29.99 ^6,8,9,10^	5.00	−0.60	1.09
Macedonia	1357	33.93 ^1,2,3,4,5,7,9,10^	4.78	−1.16	2.29
Poland	1728	30.84 ^3,6,7,8^	4.51	−0.39	1.57
Slovakia	1149	30.68 ^3,6,7,8^	5.11	−0.56	1.06
Total	14,590	30.78	5.34	−0.60	1.18

Multiple comparisons with Bonferroni; the mean difference is significant at the 0.05 level; ^1^ Austria, ^2^ Belgium, ^3^ Czechia, ^4^ England, ^5^ Estonia, ^6^ Finland, ^7^ Germany, ^8^ Macedonia, ^9^ Poland, ^10^ Slovakia.

**Table 2:** Correlation coefficients between health literacy total score (10–40), family affluence (0–13), and self-rated health (1–4).

	r(HL, FAS)	r(HL, SRH)	r(SRH, FAS)
Austria	0.21 ***	0.18 ***	0.11 ***
Belgium (Flemish)	0.12 **	0.25 ***	0.20 **
Czechia	0.11 ***	0.27 ***	0.13 ***
England	0.17 ***	0.23 ***	0.17 ***
Estonia	0.12 ***	0.23 ***	0.16 ***
Finland	0.15 ***	0.24 ***	0.10 **
Germany	0.11 ***	0.12 ***	0.13 ***
Macedonia	0.11 ***	0.22 ***	0.07 *
Poland	0.12 ***	0.16 ***	0.03
Slovakia	0.15 ***	0.23 ***	0.05
Total	0.09 ***	0.24 ***	0.08 ***

*** *p* < 0.001; ** *p* < 0.01; * *p* < 0.05.

**Table 3:** Path model with direct and indirect effects between gender, family affluence, health literacy, and self-rated health (standardized Beta coefficients).

	Standardized Betas of Direct Effect							Standardized Betas of Indirect Effects	
	Model 3	Model 4	Model 5					Model 5	
	Gender→SRH	FAS→SRH	Gender→HL	FAS→HL	HL→SRH	Gender→SRH	FAS→SRH	Gender→HL→SRH	FAS→HL→SRH
Austria	−0.15 ***	0.06	−0.04	0.16 ***	0.17 ***	−0.14 ***	0.04	−0.01	0.03 ***
Belgium (Flemish)	−0.18 ***	0.14 ***	−0.04	0.10 ***	0.23 ***	−0.18 ***	0.13 ***	−0.01	0.02 ***
Czechia	−0.12 ***	0.08 ***	0.00	0.09 ***	0.26 ***	−0.13 ***	0.07 ***	0.00	0.02 ***
England	−0.09 *	0.17 ***	0.03	0.16 ***	0.22 ***	−0.11 **	0.14 ***	0.01	0.04 ***
Estonia	−0.13 ***	0.12 ***	0.10 **	0.12 ***	0.25 ***	−0.18 ***	0.10 ***	0.02 **	0.03 ***
Finland	−0.14 ***	0.10 **	0.06	0.13 ***	0.25 ***	−0.14 ***	0.06 *	0.02 *	0.03 **
Germany	−0.13 ***	0.08 **	0.04	0.10 ***	0.12 ***	−0.13 ***	0.07 *	0.00	0.01 *
Macedonia	−0.13 ***	0.05 *	0.06 *	0.09 ***	0.24 ***	−0.14 ***	0.03	0.02 *	0.02 **
Poland	−0.14 ***	0.05	0.10 ***	0.10 ***	0.18 ***	−0.16 ***	0.03	0.02 **	0.02 **
Slovakia	−0.16 ***	0.02	0.06	0.12 ***	0.25 ***	−0.16 ***	−0.01	0.01	0.03 ***

HL = health literacy, FAS = family affluence, SRH = self-rated health. *** *p* < 0.001; ** *p* < 0.01; * *p* < 0.05.

## Supplementary Materials

The following are available online at https://www.mdpi.com/1660-4601/17/10/3543/s1.
