# Supplementary material for: A Comparative Study on Adolescents’ Health Literacy in Europe: Findings from the HBSC Study
Source: Int J Environ Res Public Health. 2020 May 19;17(10):3543. doi: 10.3390/ijerph17103543 (PMC7277198; doi:10.3390/ijerph17103543)
Supplement: Supplementary file 1 [file ijerph-17-03543-s001.pdf]

Supplementary File 1. Descriptive statistics of gender, family affluence, and self-rated health by country.

Country	Gender			FAS			SRH			
	Boys N (%)	Girls N (%)	Total N (%)	Lowest 20 pct N (%)	Medium 60 pct N (%)	Highest 20 pct N (%)	Poor N (%)	Fair N (%)	Good N (%)	Execellent N (%)
**Austria**	616 (45.7)	731 (54.3)	1374 (100.0)	204 (15.7)	830 (63.9)	265 (20.4)	23 (1.7)	159 (11.9)	724 (54.1)	433 (32.3)
**Belgium (Flemish)**	711 (48.7)	749 (51.3)	1460 (100.0)	269 (18.7)	890 (61.8)	282 (19.6)	22 (1.5)	252 (17.3)	864 (59.4)	316 (21.7)
**Czechia**	1934 (50.7)	1880 (49.3)	3814 (100.0)	806 (21.5)	2396 (63.9)	547 (14.6)	70 (1.8)	582 (15.3)	2352 (61.7)	807 (21.2)
**England**	432 (50.1)	431 (49.9)	863 (100.0)	168 (19.9)	502 (59.5)	173 (20.5)	19 (2.2)	112 (13.2)	490 (57.6)	229 (26.9)
**Estonia**	759 (49.2)	783 (50.8)	1542 (100.0)	295 (19.4)	913 (60.0)	313 (20.6)	16 (1.0)	265 (17.2)	801 (52.0)	457 (29.7)
**Finland**	531 (49.3)	545 (50.7)	1076 (100.0)	222 (21.8)	604 (59.3)	192 (18.9)	28 (2.6)	182 (17.1)	644 (60.5)	211 (19.8)
**Germany**	659 (43.4)	858 (56.6)	1517 (100.0)	247 (16.5)	1024 (68.2)	230 (15.3)	21 (1.4)	195 (12.9)	825 (54.7)	468 (31.0)
**Macedonia**	736 (49.2)	760 (50.8)	1496 (100.0)	265 (18.6)	838 (58.8)	323 (22.7)	8 (0.5)	58 (3.9)	400 (26.8)	1024 (68.8)
**Poland**	852 (47.8)	929 (52.2)	1781 (100.0)	240 (13.8)	1251 (71.7)	254 (14.6)	70 (3.9)	297 (16.7)	1114 (62.8)	294 (16.6)
**Slovakia**	703 (54.4)	590 (45.6)	1293 (100.0)	222 (22.1)	545 (54.3)	237 (23.6)	20 (1.6)	160 (12.4)	767 (59.5)	341 (26.5)

FAS = family affluence scale; SRH = self-rated health
